# Risk Factors for Early Recurrence of Gallstones in Patients Undergoing Laparoscopy Combined With Choledochoscopic Lithotomy: A Single-Center Prospective Study

**DOI:** 10.3389/fsurg.2021.759390

**Published:** 2021-11-24

**Authors:** Bo Wang, Anhua Huang, Min Jiang, Haidong Li, Wenqing Bao, Kan Ding, Zhaoyan Jiang, Gang Zhao, Hai Hu

**Affiliations:** Center of Gallbladder Diseases, Shanghai East Hospital, Tongji University School of Medicine, Shanghai, China

**Keywords:** gallstone, laparoscopy combined with choledochoscope lithotomy, risk factors, recurrence, follow-up

## Abstract

**Objective:** For patients with gallstones, laparoscopy combined with choledochoscopic lithotomy is a therapeutic surgical option for preservation rather than the removal of the gallbladder. However, postoperative recurrence of gallstones is a key concern for both patients and surgeons. This prospective study was performed to investigate the risk factors for early postoperative recurrence of gallstones.

**Methods:** The clinical data of 466 patients were collected. Each patient was followed up for up to 2 years. The first follow-up visit occurred 4 months after the operation, and a follow-up visit was carried out every 6 months thereafter. The main goal of each visit was to confirm the presence or absence of gallbladder stones. The factors associated with gallstone recurrence were analyzed by univariate analysis and Cox regression.

**Results:** In total, 466 eligible patients were included in the study, and 438 patients (180 men and 258 women) completed the 2-year postoperative follow-up. The follow-up rate was 94.0%. Recurrence of gallstones was detected in 5.71% (25/438) of the patients. Univariate analysis revealed five risk factors for the recurrence of gallstones. Multivariate Cox regression analysis showed that multiple gallstones, a gallbladder wall thickness of ≥4 mm, and a family history of gallbladder stones were the three predictive factors for postoperative recurrence of gallstones (*P* < 0.05).

**Conclusion:** The overall 2-year recurrence rate of gallstones after the operation was 5.71%. Multiple gallstones, a gallbladder wall thickness of ≥4 mm, and a family history of gallstones were the three risk factors associated with early postoperative recurrence of gallstones.

## Background

Gallstone disease is common worldwide. With the recent rapid economic developments and trends toward a Westernized lifestyle, eating habits, and dietary structure, the incidence of gallstones has reached >10% in the general population (1). Gallstones are associated with potential hazards that may lead to acute cholecystitis or related severe complications that may become life-threatening.

Laparoscopic cholecystectomy is considered the gold standard treatment for gallstones. However, laparoscopic cholecystectomy-related bile duct injuries and bile leakage are detrimental, although they occur at a very low rate (2). Moreover, cholecystectomy is reportedly associated with postoperative abnormalities such as dyspepsia, diarrhea, gastroesophageal reflux, and an increased incidence of choledocholithiasis (3). Long-term disadvantages of cholecystectomy have also been reported, including suppression of the immune system (4, 5), hyperlipidemia (6), liver steatosis (7), and an increased risk of colon cancer (8–10). These complications have gained the attention of both doctors and patients, who have recognized the importance of the gallbladder in maintaining physiological functions (11).

It is therefore rational to explore the application of laparoscopy combined with choledochoscopic lithotomy as a surrogate therapeutic option for subgroups of patients with gallstones who still have normal gallbladder function. However, the risk of postoperative gallstone recurrence remains controversial (12). Some early studies (13, 14) showed that the 5-year recurrence rate of gallstones was >30% after laparoscopy combined with choledochoscopic lithotomy. However, these studies lacked strict selection criteria for patients suitable for this operation; in particular, whether gallbladder function was normal had not been considered. Furthermore, no postoperative medical interventions were applied. With surgeons' accumulated experience in this area and the development of a proposed clinical guideline in recent decades, laparoscopy combined with choledochoscopic lithotomy has been gradually proven to be safe and effective with a relatively low rate of gallstone recurrence (15, 16). A recent retrospective study (17) showed that the overall recurrence rate of cholecystolithiasis was 9.66% among 145 patients after choledochoscopic gallbladder-preserving surgery with a median follow-up of 36 months, and the estimated 5-year recurrence rate for all patients was 12.5%. Our research group found that most cases of gallstone recurrence occurred within 2 years postoperatively, in line with a previous report (18). Another study (19) showed that the overall recurrence rate of gallbladder stones was 9.3% with a median follow-up of 59 months. Most recurrences occurred within 2 years; the recurrence rate was 7.6%. Therefore, it is important to investigate the risk factors associated with the early recurrence of gallstones within 2 years after surgery.

In this prospective study, 438 patients underwent laparoscopy combined with choledochoscopic lithotomy and were followed up for 2 years after the operation. We found that the recurrence rate of gallstones was 5.71%. The factors associated with gallstone recurrence included multiple gallstones, a gallbladder wall thickness of ≥4 mm, and a family history of gallbladder stones.

## Patients and Methods

### Inclusion and Exclusion Criteria

**Figure 1:**
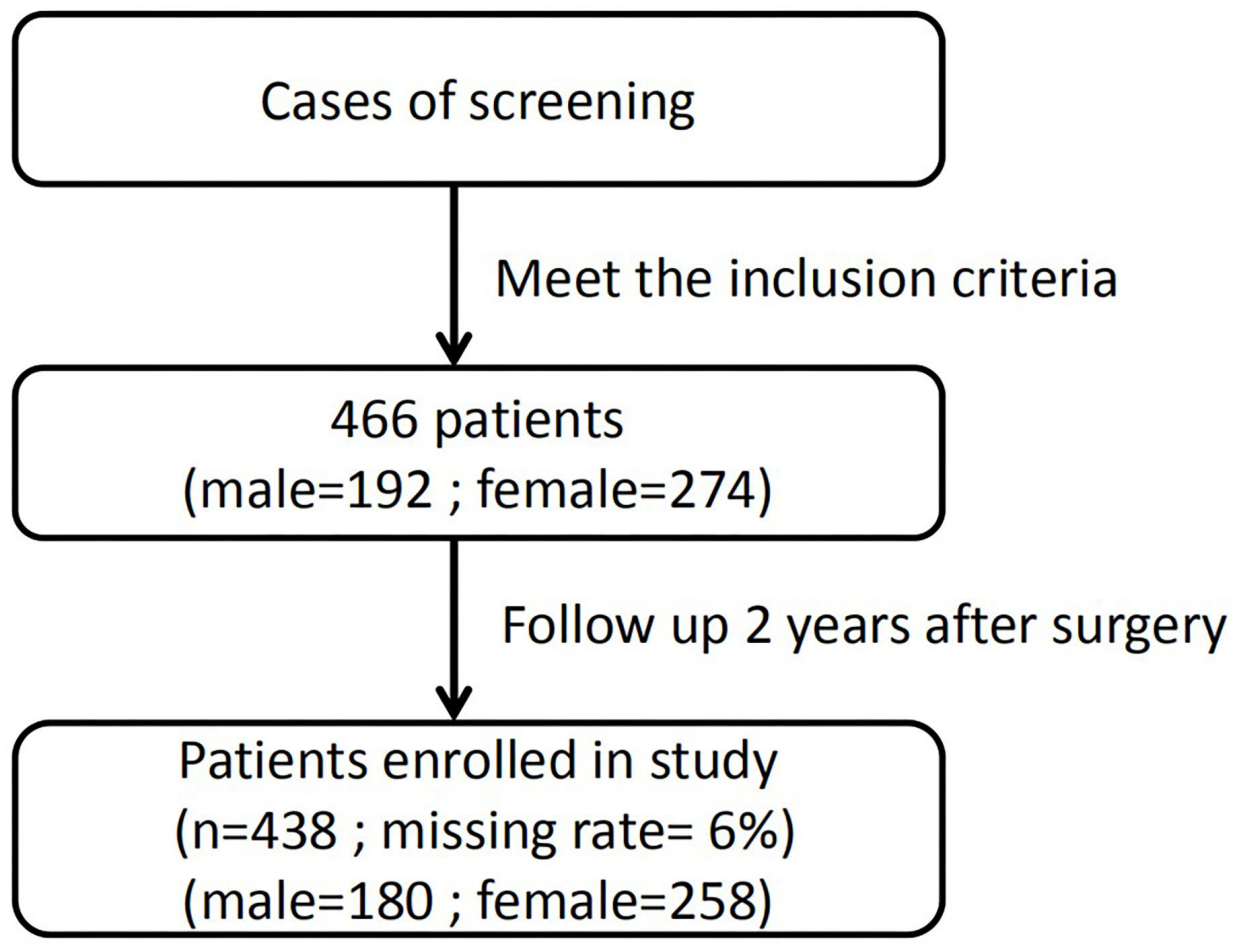
Study flowchart.

All patients in this study underwent laparoscopy combined with choledochoscopic lithotomy at the Center of Gallbladder Diseases of Shanghai East Hospital, Tongji University School of Medicine from March 1, 2015, to March 1, 2017. Figure 1 shows the flow chart of this study.

The inclusion criteria for the operation were as follows: (1) the patients who had the gallstones were somewhat symptomatic. Most patients had a dull pain in the upper abdomen or right upper abdomen when eating too much, eating high-fat foods, working stress or not having a good rest, or have full discomfort, belching, hiccups, etc.; (2) the presence of gallstones as diagnosed by ultrasonography and a gallstone volume of no more than half the fasting volume (FV) of the gallbladder; (3) evaluation of the gallbladder contractility function by B-type ultrasonography in the fasting state and 90 min after the ingestion of high-fat food (two fried eggs) as previously described (20), with a >50% calculated ejection fraction of the gallbladder; (4) no history of upper abdominal surgery; and (5) the patient's strong willingness to preserve the gallbladder with awareness of the possibility of gallstone recurrence after the operation.

The exclusion criteria were as follows: (1) any complications, including acute cholecystitis, acute cholangitis, biliary pancreatitis, or gallbladder atrophy, as identified by B-type ultrasonography or intraoperative examination; (2) partial/complete cystic duct obstruction, common bile duct stones, ulcerative colitis, or Crohn's disease; (3) suspicion of gallbladder malignancy; (4) a <50% calculated ejection fraction of the gallbladder; (5) conversion to cholecystectomy during the operation because of gallbladder conditions unsuitable for laparoscopy combined with choledochoscopic lithotomy, such as adhesion between the gallbladder and other tissue, gallbladder adenomyomatosis, or a folded gallbladder; (6) lack of postoperative follow-up data or inability to perform follow-up for 2 years; and (7) lack of treatment with tauroursodeoxycholic acid (TUDCA) at 500 mg/day for at least 6 months.

### Clinical Data

Clinical data, including sex, age, body mass index (BMI), family history, presence of diabetes, dietary habits, physical activity, duration of gallstone disease, and family history of gallstone disease, were collected. On the morning of admission, venous blood was collected for the measurement of serum biochemical variables such as liver enzymes, lipids, glucose, and electrolytes. Data on the number of gallstones, thickness of the gallbladder wall, and gallbladder contractility function were also collected. The patients' dietary habits were graded as fatty with an irregular pattern (eating high-fat foods, such as fried foods, butter, cheese, bacon, and ice cream; not eating breakfast, eating one meal and forgetting the next meal, or waiting a long interval between two meals) or light with a regular pattern. Physical activity was graded as yes (exercise such as jogging, yoga, or swimming at least twice a week for no <2 h each time) or no (no regular exercise; i.e., sedentary). Data on the gallbladder contractility function and thickness of the gallbladder wall were obtained by B-type ultrasonography as previously described (20). The gallbladder volume was measured in the fasting state and 90 min after the ingestion of high-fat food (two fried eggs) and calculated by the following formula: volume = π/6 × height × width × length (mL). The FV was designated from the residual volume (RV). The emptying fraction was calculated as follows: (FV – RV)/FV × 100%. The gallstones were collected during the operation and counted.

### Operative Procedure

Under general anesthesia, all patients were maintained in the supine reverse Trendelenburg position with the table tilted downward to the patient's left, and carbon dioxide pneumoperitoneum was established with an intraperitoneal pressure of ~13–14 mmHg. A laparoscope was inserted through an umbilical trocar. After laparoscopic monitoring, one 5-mm trocar was placed in the right subcostal region, which was the body surface projection of the gallbladder fundus (where the choledochoscope was introduced); another 5-mm trocar was placed below the xiphoid (where the surgical instruments were introduced). A piece of dry gauze was placed around the gallbladder, and a small incision was made at the bottom of the gallbladder fundus according to the size of the stones. The stones were extracted with a grasping basket, removed from the gallbladder, and placed in a specimen bag. When the stones had been completely removed, the choledochoscope was placed again. The bile flow was observed refluxing from the gallbladder tube, and the cystic duct patency was confirmed. No bleeding or residual stones were present in the gallbladder lumen after the procedure, and the incision was closed using a running suture of 4/0 Vicryl Plus (ETHICON Johnson & Johnson, New Brunswick, NJ, United States). The specimen bag and gauze were removed through the right-side 10-mm working channel. Finally, the operative field was washed repeatedly with saline and suctioned. In the absence of active bleeding and bile leakage, no drainage tube was routinely placed. The trocars were pulled out, and the ports were closed with stitches. The instruments used in this study included a laparoscope (Storz, Tuttlingen, Germany), a soft choledochoscope (Olympus, Tokyo, Japan), a grasping basket for stone removal, and a biopsy clamp. All the surgical procedures in this study were completed by the same attending surgeon and his team.

### Postoperative Management

All patients were discharged the second day after the operation with no complications. The day after the operation, all patients started treatment with TUDCA at a dose of 5 mg/kg orally once a day, and the therapy was continued for at least 6 months. The patients' liver function was monitored at 3-month intervals to check for adverse effects of the drug. The patients were encouraged to implement a low-fat diet and proper exercise in their daily life according to the instructions prepared by the healthcare center.

### Patient Follow-Up

All patients were followed up for 2 years, and the findings of the follow-up visits were recorded. The first follow-up visit occurred 4 months after the laparoscopy combined with choledochoscopic lithotomy, and additional follow-up visits occurred every 6 months thereafter. During each follow-up visit, the patients were clinically examined. B-type ultrasonography of the gallbladder was performed. Gallstone recurrence was defined as the detection of any echogenic object with an acoustic shadow or gravity dependence (sludge) in the gallbladder.

### Statistical Analysis

Continuous variables are expressed as mean ± SD, and categorical variables are expressed as number and percentage. Differences between groups were compared by the *t*-test or chi-square test. Univariate analysis and multivariate Cox regression analysis were performed to explore the risk factors associated with gallstone recurrence. A two-sided *P*-value of < 0.05 was considered statistically significant. The predicted risk factors for early gallbladder stone recurrence after laparoscopy combined with choledochoscopic lithotomy were also analyzed by Kaplan–Meier survival analysis. All statistical analyses were performed using SPSS 23.0 software (IBM Corp., Armonk, NY, United States).

## Results

### Patients' Characteristics

From March 1, 2015, to March 1, 2017, 466 patients underwent laparoscopy combined with choledochoscopic lithotomy. Twenty-eight patients dropped out during the follow-up period and were excluded. Thus, 438 patients were analyzed in this study, including 180 men and 258 women (men: women ratio of 1.00:1.43). Within this cohort, the follow-up rate was 93.99%. The mean age at admission was 42.5 ± 13.1 years (range, 22–75 years).

The mean follow-up time after surgery was 23.3 ± 2.9 months. Twenty-five patients had disease recurrence (recurrence group), with a 5.71% overall recurrence rate. Of the 25 patients who developed recurrence, 16 (64%) underwent cholecystectomy and 9 (36%) received conservative treatment.

Those who had recurred gallstones (64%) still have some clinical symptoms related to stones or the diameter of the stones is >1.0 cm or the number of recurring stones is >1. However, for the rest of the patients (36%), the number of recurring stones is only 1, and the diameter of the stones is <1.0 cm; therefore, they are not subjected to laparoscopic cholecystectomy yet. These patients are regularly and intensively followed up and still treated with TUDCA. The baseline characteristics of all patients are shown in Table 1.

**Table 1:** Patients' general clinical data (*n* = 438).

**General data**		**Recurrence**	**No recurrence**
Cases (men/women)		12/13	168/246
Age (y)		41.44 ± 13.6	43.75 ± 13.9
BMI (Kg/m^2^)		23.93 ± 3.35	23.58 ± 3.66
Dietary habit (fatty diet)	fatty	13 (52%)	142 (34.38%)
Physical activity (yes)	yes	20 (80%)	317 (76.76%)
Duration of the gallstone disease (*n*)	≥2year	10	220
	<2year	15	193
Number of gallstones (*n*)	single	4	196
	multiple	21	217
EF of gallbladder (%)		0.57 ± 0.32	0.63 ± 0.31
Thickness of gallbladder wall (mm)		4.56 ± 4.12	3.27 ± 1.62
History of diabetes (yes)	yes	3 (12%)	25 (6.05%)
Presence of family history of	yes	5 (20%)	6 (1.45%)
gallstones (yes)			
TC (mmol/L)		4.02 ± 0.81	4.30 ± 1.32
TG (mmol/L)		1.72 ± 2.44	1.33 ± 0.92

*Data are expressed as n (%) or mean ± SD.*

*BMI, body mass index; EF, ejection fraction; TC, total cholesterol; TG, triglycerides*.

Age and BMI did not differ between the two groups. Two hundred (45.7%) patients had a single gallstone, whereas 238 (55.3%) patients had multiple stones. The ejection fraction of the gallbladder was comparable between the recurrence group (0.57% ± 0.32%) and the non-recurrence group (0.63% ± 0.31%) (*P* > 0.05).

### Analysis of Factors Associated With Recurrence of Gallstones

We first performed a univariate analysis for the association of factors with the postoperative recurrence of gallstones. We found that multiple gallbladder stones (odds ratio [OR], 0.227; 95% confidence interval [CI], 0.077–0.671; *P* = 0.004), a gallbladder wall thickness of ≥4 mm (OR, 4.726; 95% CI, 1.452–5.385; *P* = 0.020), a family history of gallbladder stones (OR, 16.958; 95% CI, 4.768–60.318; *P* = 0.000), a high serum total cholesterol level (OR, 4.157; 95% CI, 1.692–10.213; *P* = 0.003), and a high serum triglyceride level (OR, 35.270; 95% CI, 12.597–98.748; *P* = 0.000) were associated with the recurrence of gallstones (Table 2). Sex, age, BMI, dietary habits, physical activity, duration of the gallstone disease, gallbladder contractility function, and diabetes were not associated with the recurrence of gallstones (Table 2).

**Table 2:** Results of chi-square test of postoperative gallbladder stone recurrence.

**Risk factors**	**OR**	**95% CI**	***P***
Sex	1.639	0.728–3.691	0.229
Age	1.099	0.365–3.313	0.867
BMI	1.683	0.551–5.135	0.557
Dietary habit	2.067	0.919–4.650	0.074
Physical activity	1.211	0.443–3.313	0.708
Duration of the gallstone disease	0.585	0.257–1.332	0.197
Number of gallstones (single vs. multiple)	0.227	0.077–0.671	**0.004**
EF of gallbladder	0.917	0.373–2.251	0.850
Thickness of gallbladder wall	4.726	1.452–15.385	**0.020**
History of diabetes	2.116	0.593–7.553	0.448
Presence of family history of gallstones	16.958	4.768–60.318	**0.000**
TC	4.157	1.692–10.213	**0.003**
TG	35.270	12.597–98.748	**0.000**

*OR, odds ratio; CI, confidence interval; BMI, body mass index; EF, ejection fraction; TC, total cholesterol; TG, triglycerides*.

We then performed a multivariate Cox regression analysis. The results showed that multiple gallbladder stones (hazard ratio [HR], 4.321; 95% CI, 1.478–12.631; *P* = 0.007), a gallbladder wall thickness of ≥4 mm (HR, 4.289; 95% CI, 1.451–12.677; *P* = 0.008), and a family history of gallbladder stones (HR, 11.878; 95% CI, 4.375–32.251; *P* = 0.000) remained as independent factors associated with gallstone recurrence (Table 3).

**Table 3:** Results of Cox regression analysis of postoperative gallbladder stone recurrence.

**Risk factors**	**HR**	**95% CI**	***P***
Number of gallstones	4.321	1.478–12.631	**0.007**
Thickness of gallbladder wall	4.289	1.451–12.677	**0.008**
Presence of family history of gallstones	11.878	4.375–32.251	**0.000**

*HR, hazard ratio; CI, confidence interval*.

### Kaplan–Meier Analysis

**Figure 2:**
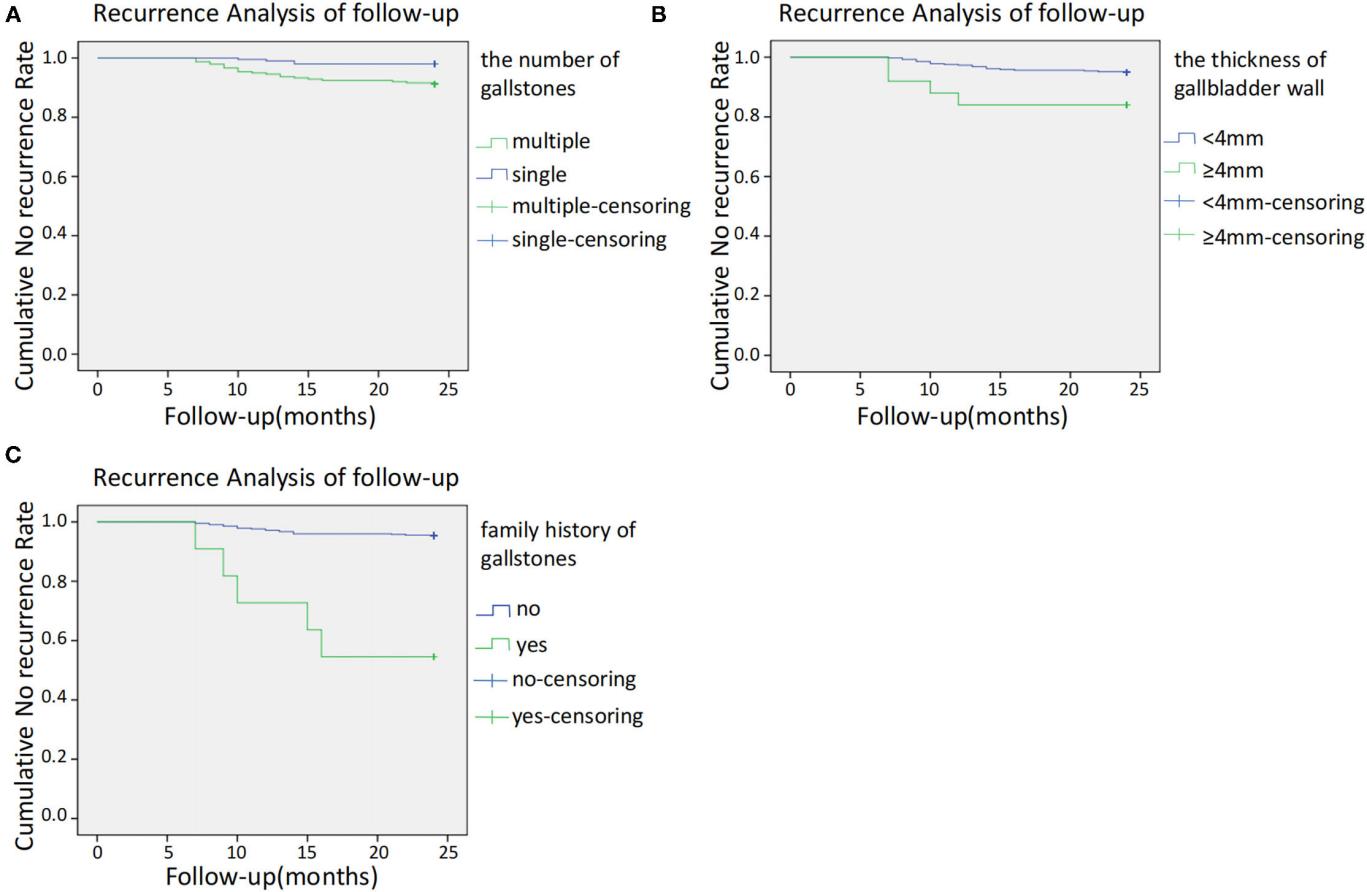
**(A)** Kaplan–Meier cumulative no recurrence rate according to the number of gallstones. **(B)** Kaplan–Meier cumulative no recurrence rate according to a thickness of gallbladder wall. **(C)** Kaplan–Meier cumulative no recurrence according to family history.

We further stratified the patients according to the number of gallstones, gallbladder thickness, and presence of a family history of gallbladder stones and performed a Kaplan–Meier analysis. The recurrence rate among patients with a single gallbladder stone was 2% (4/200), and that among patients with multiple gallbladder stones was 8.8% (21/238). The mean recurrence time in patients with a single gallbladder stone was 23.77 ± 0.12 months, which was significantly longer than that in patients with multiple gallbladder stones (22.95 ± 0.24 months) (χ^2^ = 9.42, *P* = 0.002) (Figure 2A).

The recurrence rate in patients with a gallbladder wall thickness of ≥4 and <4 mm was 20% (4/20) and 5.02% (21/418), respectively. Patients with a gallbladder wall thickness of ≥4 mm had a significantly earlier recurrence time (21.60 ± 1.11 months) than those with a gallbladder wall thickness of <4 mm (23.43 ± 0.135 months) (χ^2^ = 5.877, *P* = 0.015) (Figure 2B).

The recurrence rate in patients with and without a family history of gallbladder stones was 45.45% (5/11) and 4.68% (20/427), respectively. The recurrence time in patients with a family history of gallbladder stones (18.273 ± 2.021 months) was significantly earlier than that in patients without a family history (23.457 ± 0.132 months) (χ^2^ = 41.013, *P* = 0.000) (Figure 2C).

## Discussion

The rationale of laparoscopy combined with choledochoscopic lithotomy is to preserve the physiological function of the gallbladder. Clinicians' major concern is the postoperative recurrence of gallbladder stones. Thus, it is of great importance to clarify the risk factors associated with gallstone recurrence. In this prospective study of 438 patients, we found that the overall recurrence rate of gallstones within 2 years postoperatively was 5.71% (25/438). Furthermore, we, for the first time, identified three independent risk factors for early recurrence (within 2 years postoperatively) of gallstones: multiple gallbladder stones, a gallbladder wall thickness of ≥4 mm, and a family history of gallbladder stones.

With respect to the number of gallstones, the recurrence rate was obviously higher in patients with multiple gallstones (8.8%) than in those with a single gallstone (2.0%). Such a high recurrence rate is mainly due to the persistence of pathogenetic factors responsible for gallstone formation or the presence of predisposing conditions (21). The reason for this difference may be that patients with multiple gallstones reportedly have a faster nucleation time than patients with a single gallstone (22). Another possibility is that the wall of a gallbladder with multiple stones is more likely to be subjected to injuries from the stimulus of the gallstones, facilitating cholestasis and the recurrence of gallbladder stones (23). Furthermore, in patients with multiple gallstones, the possibility of residual minute stones within the gallbladder cannot be completely excluded. This reminds surgeons to clear the gallbladder as much as possible during choledochoscopy.

The normal gallbladder is usually flat with a single layer of columnar epithelial cells. Thickening of the gallbladder wall may be due to the proliferation of the epithelium and submucosal inflammation during the process of gallstone formation. Thickening of the gallbladder wall may also affect the smooth muscle contractile function of the gallbladder, leading to a vicious circle that promotes gallstone formation (24, 25).

The histopathologic alterations in the gallbladder wall that indicate inflammation include edema, increased gallbladder wall thickness, and the presence of inflammatory cells. These changes can promote the production and secretion of mucin glycoproteins (26). Cholesterol monohydrate crystals nucleate in the gelled mucin glycoprotein scaffolding on the gallbladder wall (27–30). Mucin genes that are expressed in human biliary epithelia include MUC1, MUC2, MUC3, MUC4, MUC5AC, MUC5B, and MUC6 (31). Mucin gel accumulation appears to be important for the formation of cholesterol gallstones in humans and animal models (32–36).

Lim et al. (37) found a significant association between the muscle-to-fibrosis thickness ratio of the gallbladder wall and a low ejection fraction. The thickness of the gallbladder wall is reportedly an indicator of increasing inflammation (38), and thickened gallbladder walls contribute to a reduced ejection fraction of the gallbladder (39). Furthermore, bile stasis and gallbladder obstruction lead to ischemic and inflammatory changes in the gallbladder wall, finally resulting in gallstone recurrence.

A family history of gallbladder stones is another important risk factor for recurrence as verified by both research and clinical practice (40). The phenomenon of family aggregation of gallstone disease is possible because of shared genetic susceptibility to gallstones, similar eating habits, and similar lifestyle and geographic factors. In the present study, the recurrence rate of gallstones was significantly higher in patients with a family history of gallstones (5/11, 45.45%) than in those without a family history of gallstones (20/427, 4.68%) (*P* < 0.05). The relevant genes for gallstone disease in humans reported to date include the apolipoprotein A (ApoA) genes (41), apolipoprotein B (ApoB) genes (42), ATP-binding cassette subfamily G (ABCG) transporter genes (43), and the cholecystokinin A receptor (CCK-AR) genes (44). The present study suggests that patients with certain genetic susceptibility might have an increased risk of gallstone recurrence; laparoscopy combined with choledochoscopic lithotomy should be performed with caution in these patients, or they should be intensively followed up after the operation.

The total gallstone recurrence rate was very low (5.71%) in the present study. This is partly because of the strict inclusion criteria and selection of appropriate candidates before the operation. A previous study (45) showed that throughout 25 years of follow-up, patients with gallbladder contractility function of >30% had a low postoperative recurrence rate; in our study, however, a >50% calculated ejection fraction of the gallbladder was a strict inclusion criterion. Laparoscopy combined with choledochoscopic lithotomy breaks the limitations of the operation itself by a careful and gentle operation, complete removal of the stones, comprehensive inspection, and a careful intraoperative evaluation of the patients' characteristics and gallbladder condition. Postoperative education for patients is important to ensure that they maintain a healthy diet, reasonable exercise and lifestyle, and regular medication such as TUDCA for an adequate amount of time.

## Conclusion

In this prospective study, we showed that the total gallstone recurrence rate was 5.71% for patients with gallstones who underwent laparoscopy combined with choledochoscopic lithotomy. The predicted risk factors for early postoperative gallbladder stone recurrence within 2 years after laparoscopy combined with choledochoscopic lithotomy were multiple gallbladder stones, a gallbladder wall thickness of ≥4 mm, and a family history of gallbladder stones.

## Data Availability Statement

The original contributions presented in the study are included in the article/supplementary material, further inquiries can be directed to the corresponding authors.

## Ethics Statement

The studies involving human participants were reviewed and approved by the Ethics Committee of Shanghai East Hospital of Tongji University. The patients/participants provided their written informed consent to participate in this study.

## Author Contributions

BW, AH, and MJ: acquisition of data and drafting the manuscript. ZJ, GZ, and HH: conception and design of study. HL, WB, and KD: analysis and/or interpretation of data. BW, AH, MJ, ZJ, and HH: revising the manuscript critically for important intellectual content. All authors contributed to the article and approved the submitted version.

## Funding

This work was supported by the National Natural Science Foundation of China (Grant Number 91570577) and the Key Specialty Construction Project of Pudong Health and Family Planning Commission of Shanghai (Grant Number PWZzk2017-10).

## Conflict of Interest

The authors declare that the research was conducted in the absence of any commercial or financial relationships that could be construed as a potential conflict of interest.

## Publisher's Note

All claims expressed in this article are solely those of the authors and do not necessarily represent those of their affiliated organizations, or those of the publisher, the editors and the reviewers. Any product that may be evaluated in this article, or claim that may be made by its manufacturer, is not guaranteed or endorsed by the publisher.

## References

[1] Stinton LM Myers RP Shaffer EA. Epidemiology of gallstones. Gastroenterol Clin North Am. (2010) 39:157–69. 10.1016/j.gtc.2010.02.003 20478480

[2] Shirah BH Shirah HA Zafar SH Albeladi KB. Clinical patterns of postcholecystectomy syndrome. Ann Hepatobiliary Pancreat Surg. (2018) 22:52–7. 10.14701/ahbps.2018.22.1.52 29536056 PMC5845611

[3] Ahmad DS Faulx A. Management of postcholecystectomy biliary complications: a narrative review. Am J Gastroenterol. (2020) 115:1191–8. 10.14309/ajg.0000000000000704 32483004

[4] Sarashina-Kida H Negishi H Nishio J Suda W Nakajima Y Yasui-Kato M. Gallbladder-derived surfactant protein D regulates gut commensal bacteria for maintaining intestinal homeostasis. Proc Natl Acad Sci USA. (2017) 114:10178–83. 10.1073/pnas.1712837114 28878025 PMC5617315

[5] Reynoso-Paz S Coppel RL Mackay IR Bass NM Ansari AA Gershwin ME. The immunobiology of bile and biliary epithelium. Hepatology. (1999) 30:351–7. 10.1002/hep.510300218 10421640

[6] Malik AA Wani ML Tak SI Irshad I Ul-Hassan N. Association of dyslipidaemia with cholilithiasis and effect of cholecystectomy on the same. Int J Surg. (2011) 9:641–2. 10.1016/j.ijsu.2011.08.003 21933723

[7] Ruhl CE Everhart JE. Relationship of non-alcoholic fatty liver disease with cholecystectomy in the US population. Am J Gastroenterol. (2013) 108: 952–8. 10.1038/ajg.2013.70 23545713

[8] Shao T Yang Y-X. Cholecystectomy and the risk of colorectal cancer. Am J Gastroenterol. (2005) 100: 1813–20. 10.1111/j.1572-0241.2005.41610.x 16086719

[9] Chen YK Yeh JH Lin CL Peng CL Sung FC Hwang IM. Cancer risk in patients with cholelithiasis and after cholecystectomy: a nationwide cohort study. J Gastroenterol. (2014) 49:923–31. 10.1007/s00535-013-0846-6 23807230

[10] Zhang Y Liu H Li L Ai M Gong Z He Y. Correction: cholecystectomy can increase the risk of colorectal cancer: a meta-analysis of 10 cohort studies. PLoS ONE. (2018) 13:e0191587. 10.1371/journal.pone.0191587 29342205 PMC5771615

[11] Housset C Chrétien Y Debray D Chignard N. Functions of the gallbladder. Compr Physiol. (2016) 6:1549–77. 10.1002/cphy.c150050 27347902

[12] Søreide K. Gallstone disease and cancer risk: finding the bug in the system. Gastroenterology. (2017) 152:1825–8. 10.1053/j.gastro.2017.04.028 28461193

[13] Zou YP Du JD Li WM Xiao YQ Xu HB Zheng F. Gallstone recurrence after successful percutaneous cholecystolithotomy: a 10-year follow-up of 439 cases. Hepatobiliary Pancreat Dis Int. (2007) 6:199–203. 17374582

[14] De Caluwé D Akl U Corbally M. Cholecystectomy versus cholecystolithotomy for cholelithiasis in childhood: long-term outcome. J Pediatr Surg. (2001) 36:1518–21. 10.1053/jpsu.2001.27035 11584400

[15] Hu H Huang A Zhang W Wang W Zhu J Chen BG. Gas-free single-port transumbilical laparoscopic cholecystolithotomy: preliminary report on eight cases. J Laparoendosc Adv Surg Tech A. (2011) 21:221–5. 10.1089/lap.2010.0517 21457112

[16] Kim YH Kim YJ Shin TB. Fluoroscopy-guided percutaneous gallstone removal using a 12-Fr sheath in high-risk surgical patients with acute cholecystitis. Korean J Radiol. (2011) 12:210–5. 10.3348/kjr.2011.12.2.210 21430938 PMC3052612

[17] Du QC Wang YY Hu CL Zhou Y. Reconsideration of indications for choledochoscopic gallbladder-preserving surgery and preventive measures for postoperative recurrence of gallstones. Wideochir Inne Tech Maloinwazyjne. (2020) 15:87–96. 10.5114/wiitm.2019.88647 32117490 PMC7020701

[18] Roqués JL Pruneda RR Sánchez J Girón O Aranda MJ Trujillo A. Cholecystolithotomy: first middle-long term results of our series. Cir Pediatr. (2009) 22:153–6. 19957865

[19] Qu Q Chen W Liu X Wang W Hong T Liu W. Role of gallbladder-preserving surgery in the treatment of gallstone diseases in young and middle-aged patients in China: results of a 10-year prospective study. Surgery. (2020) 167:283–9. 10.1016/j.surg.2019.09.001 31606197

[20] Dodds WJ Groh WJ Darweesh RM Lawson TL Kishk SM Kern MK. Sonographic measurement of gallbladder volume. AJR Am J Roentgenol. (1985) 145:1009–11. 10.2214/ajr.145.5.1009 3901703

[21] Portincasa P. Moschetta A, Palasciano G. Cholesterol gallstone disease. Lancet. (2006) 368:230–9. 10.1016/S0140-6736(06)69044-2 16844493

[22] Villanova N Bazzoli F Taroni F Frabboni R Mazzella G Festi D. Gallstone recurrence after successful oral bile acid treatment. A 12-year follow-up study and evaluation of long-term postdissolution treatment. Gastroenterology. (1989) 97:726–31. 10.1016/0016-5085(89)90644-6 2753332

[23] Tan YY Zhao G Wang D Wang JM Tang JR Ji ZL. A new strategy of minimally invasive surgery for cholecystolithiasis: calculi removal and gallbladder preservation. Dig Surg. (2013) 30:466–71. 10.1159/000357823 24481280

[24] Bonatti M Vezzali N Lombardo F Ferro F Zamboni G Tauber M. Gallbladder adenomyomatosis: imaging findings, tricks and pitfalls. Insights Imaging. (2017) 8:243–53. 10.1007/s13244-017-0544-7 28127678 PMC5359147

[25] Pasternak A Bugajska J Szura M Walocha JA Matyja A Gajda M. Biliary polyunsaturated fatty acids and telocytes in gallstone disease. Cell Transplant. (2017) 26:125–33. 10.3727/096368916X692717 27502173 PMC5657685

[26] Maurer KJ Carey MC Fox JG. Roles of infection, inflammation, and the immune system in cholesterol gallstone formation. Gastroenterology. (2009) 136:425–40. 10.1053/j.gastro.2008.12.031 19109959 PMC2774219

[27] Carey MC. Pathogenesis of gallstones. Am J Surg. (1993) 165:410–9. 10.1016/S0002-9610(05)80932-8 8480873

[28] Carey MC. Pathogenesis of gallstones. Recenti Prog Med. (1992) 83:379–91. 1529152

[29] Lee SP LaMont JT Carey MC. Role of gallbladder mucus hypersecretion in the evolution of cholesterol gallstones. J Clin Invest. (1981) 67:1712–23. 10.1172/JCI110209 7240416 PMC370748

[30] Wang D Q Paigen B Carey M C. Phenotypic characterization of Lith genes that determine susceptibility to cholesterol cholelithiasis in inbred mice: physical-chemistry of gallbladder bile. J Lipid Res. (1997) 38:1395–411. 10.1016/S0022-2275(20)37422-8 9254065

[31] Andrianifahanana M Moniaux N Batra SK. Regulation of mucin expression: mechanistic aspects and implications for cancer and inflammatory diseases. Biochim Biophys Acta. (2006) 1765:189–222. 10.1016/j.bbcan.2006.01.002 16487661

[32] Lammert F Wang DQ Wittenburg H Bouchard G Hillebrandt S Taenzler B. Lith genes control mucin accumulation, cholesterol crystallization, and gallstone formation in A/J and AKR/J inbred mice. Hepatology. (2002) 36:1145–54. 10.1053/jhep.2002.36821 12395324

[33] LaMont JT Smith BF Moore JR. Role of gallbladder mucin in pathophysiology of gallstones. Hepatology. (1984) 4:51S−6S. 10.1002/hep.1840040809 6546237

[34] Lee KT Liu TS. Mucin gene expression in gallbladder epithelium. J Formos Med Assoc. (2002) 101:762–8. 12517055

[35] Wang HH Afdhal NH Gendler SJ Wang DQ. Targeted disruption of the murine mucin gene 1 decreases susceptibility to cholesterol gallstone formation. J Lipid Res. (2004) 45:438–47. 10.1194/jlr.M300468-JLR200 14703511

[36] Wang HH Afdhal NH Gendler SJ Wang DQ. Evidence that gallbladder epithelial mucin enhances cholesterol cholelithogenesis in MUC1 transgenic mice. [J] Gastroenterology. (2006) 131:210–22. 10.1053/j.gastro.2006.04.011 16831603

[37] Lim JU Joo KR Won KY Lim SJ Joo SH Yang YJ. Predictor of abnormal gallbladder ejection fraction in patients with atypical biliary pain: histopathological point of view. Medicine. (2017) 96:e9269. 10.1097/MD.0000000000009269 29390484 PMC5758186

[38] Rosen M Brody F Ponsky J. Predictive factors for conversion of laparoscopic cholecystectomy. Am J Surg. (2002) 184:254–8. 10.1016/S0002-9610(02)00934-0 12354595

[39] Zhang Y Liu D Ma Q Dang C Wei W Chen W. Factors influencing the prevalence of gallstones in liver cirrhosis. J Gastroenterol Hepatol. (2006) 21:1455–8. 10.1111/j.1440-1746.2006.04465.x 16911692

[40] Andrews S. Gallstone size related to incidence of post cholecystectomy retained common bile duct stones. Int J Surg. (2013) 11:319–21. 10.1016/j.ijsu.2013.02.009 23454243

[41] Dixit M Choudhuri G Saxena R Mittal B. Association of apolipoprotein A1-C3 gene cluster polymorphisms with gallstone disease. Can J Gastroenterol. (2007) 21:569–75. 10.1155/2007/329342 17853951 PMC2657985

[42] Gong Y Zhang L Bie P Wang H. Roles of ApoB-100 gene polymorphisms and the risks of gallstones and gallbladder cancer: a meta-analysis. PLoS ONE. (2013) 8:e61456. 10.1371/journal.pone.0061456 23637837 PMC3630192

[43] Von Kampen O Buch S Nothnagel M Azocar L Molina H Brosch M. Genetic and functional identification of the likely causative variant for cholesterol gallstone disease at the ABCG5/8 lithogenic locus. Hepatology. (2013) 57:2407–17. 10.1002/hep.26009 22898925

[44] Oude Elferink RP Beuers U. Targeting the ABCB4 gene to control cholesterol homeostasis. Expert Opin Ther Targets. (2011) 15:1173–82. 10.1517/14728222.2011.607163 21801087

[45] Martínez Castaño I Ruiz Pruneda R Doménech Abellán E Aranda Garcia MJ Sánchez Morote JM Roqué Serradilla JL. Gallbladder motility and long term results in cholecystolithotomy. Cir Pediatr. (2014) 27:173–7. 26065109

